# Pushing Droplet Through a Porous Medium

**DOI:** 10.1007/s11242-021-01705-z

**Published:** 2021-10-26

**Authors:** Maciej Matyka

**Affiliations:** Faculty of Physics and Astronomy, University of Wrocław, pl. M. Borna 9, Wrocław, 50-204 Poland

**Keywords:** Porous media, Droplet, Soft body, Spring and mass system, Simulation, Tortuosity

## Abstract

I use a mechanical model of a soft body to study the dynamics of an individual fluid droplet in a random, non-wettable porous medium. The model of droplet relies on the spring–mass system with pressure. I run hundreds of independent simulations. I average droplets trajectories and calculate the averaged tortuosity of the porous domain. Results show that porous media tortuosity increases with decreasing porosity, similar to single-phase fluid study, but the form of this relationship is different.

**Supplementary Information:**

The online version contains supplementary material available at 10.1007/s11242-021-01705-z.

## Introduction

Individual fluid droplets are ubiquitous. The motion of an individual droplet is interesting from many perspectives (Bergeron and Quéré 2001). Rain droplets, for instance, may spread, bounce and splash on leaves surface (Dorr et al. 2015) which is not only beautiful but has important applications in agriculture (Lin et al. 2016). Droplets bounce (Terwagne et al. 2013), squeeze (Perazzo et al. 2018), splash (Harlow and Shannon 1967) and freeze (Schutzius et al. 2015) in many other situations. Droplets appear important in the most expressive problem of our times during pandemia. Their dispersion in air and porous media (textiles) may become key to understand and limit virus spreading (Bandiera et al. 2020; Leung et al. 2020; Maggiolo et al. 2021).

Droplets appear in many situations in nature and technological processes. Drainage of the porous medium by a single droplet applies in the inkjet printing process (Clarke et al. 2002; Staat et al. 2017), in the oil recovery industry, when oil ganglia get trapped in pores and must be extracted using vibrations (Li et al. 2005; Perazzo et al. 2018; He et al. 2019). Droplets motion at porous and textured surfaces rises many interesting scientific questions, e.g., the pancake bouncing phenomena (Liu et al. 2014; Liu and Wang 2016; Bro et al. 2016) or the icing problem (Remer et al. 2014). In meteorology, for instance, the equilibrium shape of an individual droplet is important to predict the amount of precipitation (Beard 1976; Vollmer et al. 2012). But the most meaningful example today comes from medicine and the current pandemic situation. During the exhalation (when we talk, sing, cough or just breathe), we produce thousands of droplets that may become the carrier for viruses (Shadloo-Jahromi et al. 2020). Porous media have interesting medical applications as, for instance, lungs and surgical masks have a complex structure and transport droplets every day (Dbouk and Drikakis 2020; Leung et al. 2020; Haslbeck et al. 2010). A recent study shows the role of limited micro-droplet formation in children’s lungs in lowering COVID-19 transmission rates among the young part of the population (Riediker and Morawska 2020). Here, I concentrate on separated droplets and suggest using the mechanical model for their dynamics in a complex, porous medium.

The transport of fluids in porous media depends on three main factors: porosity ($\varphi$), permeability and tortuosity.

Tortuosity is a non-dimensional physical quantity used to characterize elongation of transport paths in the fluid flow (or in diffusion) due to the existence of pores (Clennell 1997). Thus, it may improve the Karman–Kozeny equation for permeability (Koponen et al. Sep 1997) or estimate diffusion constant in porous media (Boudreau 1996). Here, I define *T* as the ratio of the average fluid flow path lengths $\langle \lambda \rangle$ to system size *L*:

1 $$\begin{aligned} T=\frac{\langle \lambda \rangle }{L}, \end{aligned}$$

where $\langle \lambda \rangle$ is the effective length of the flow particle paths and *L* is the porous medium length. Tortuosity changes with porosity in the creeping flow regime of single-phase, incompressible fluid (Koponen et al. Jul 1996; Matyka et al. 2008). It appears in electric (Zhang and Knackstedt 1995), diffusion and hydrodynamic transport processes (Ghanbarian et al. 2013; Saomoto and Katagiri 2015).

So far, not much was done to investigate multiphase tortuosity in porous media, whereas multiphase flows and related phenomena have become important for science and engineering. Recently, ganglia mobilization during imbibition was investigated experimentally using micro-particle tracking velocimetry (Zarikos et al. 2018). In general, experimental methods for droplets are expensive, relatively inaccessible and time-consuming (Bouchard and Chandra 2019). Thus, it is important to have tools for an efficient and flexible simulation of this phenomenon. The motion of individual drops at pore-scale models of porous media was already investigated using, e.g., the boundary integral methods (Rallison and Acrivos 1978; Coulliette and Pozrikidis 1998) which turned out to be inefficient. For instance, the simulation in porous media was restricted to a maximum of eighteen particles used to build porous media samples (Davis and Zinchenko 2009). Also, the lattice Boltzmann (LBM) models were used for simulation of the transport of separated fluid droplets (Zhang et al. 2019; Liu and Wang 2016; Frank and Perre 2012). Controlling LBM multiphase simulations and, in particular, measuring quantities based on the single droplet movement may be difficult, mostly due to vanishing small droplets and spurious currents (Nabavizadeh et al. 2019).

The aim of this work is to calculate multiphase, single fluid droplet tortuosity based on the transport of a single droplet in a porous domain. For this, I develop a mechanical model of a fluid droplet based on the soft body model (Matyka et al. 2008; Matyka and Ollila 2003). I simulate the transport of a single, non-wettable fluid through a complex, superhydrophobic porous medium build of randomly distributed grains. In my model, to calculate tortuosity I track the path of the center of mass of each droplet. The model may run at real-time rates and allows me to simulate falling of many droplets through the media. This allows extending the study to perform the statistical analysis of repeated numerical experiments. In particular, I will use it to calculate tortuosity versus porosity relation in a wide range of porosity and compare it to previous results obtained for single-phase flows (Matyka et al. 2008).

## The Fluid Droplet Model

I use the spring–mass system to model droplets. A similar model was used to simulate, e.g., bouncing droplet (Terwagne et al. 2013), the red-blood-cell built of a triangulated network of springs (Fedosov et al. 2014). A comparison of the spring–mass system to continuum constitutive laws for capsules at large deformations was also made (Omori et al. 2011).

The simulated porous medium material is superhydrophobic with a wetting angle 180 degrees. The parameters of the model should be such that changes in droplet shape are small enough to ensure that the droplet does not split. To keep the volume constant, the atmospheric pressure model is used, where an additional pressure force is calculated using the ideal gas law. The previous work shows that the pressurized soft body model is efficient in simulating soft objects (Matyka and Ollila 2003). The model may be two- or three-dimensional and two dimensions are used here as it is easier to visualize and control the simulation.

**Fig. 1:**
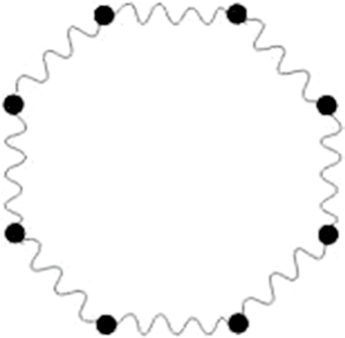
Two-dimensional soft body model is built of springs (wavy connections) and masses (filled circles). Only the boundary of the droplet is simulated. An additional pressure force is used to conserve the droplet’s volume

First, mass is distributed uniformly at droplets surface using discrete material points. Each mass is connected with its neighbor with a linear spring that represents the surface tension of the material (see Fig. 1).

Three forces act on each mass point: gravity ($\mathbf {f}=m \mathbf {g}$), linear spring force and pressure. The linear spring force between neighbors with damping reads:

2 $$\begin{aligned} \mathbf {f}_s= \left( -k_s (d - d_0) - k_d \frac{(\mathbf {v}_i-\mathbf {v}_j)\cdot \mathbf {r}_{ij}}{|\mathbf {r}_{ij}|}\right) \mathbf {r}_{ij}/|\mathbf {r}_{ij}|, \end{aligned}$$

where $k_s$ $[M/T^2]$ is the spring constant , *d* [*L*] is the distance between the *i*-th and *j*-th mass, $d_0$ [*L*] is the distance at rest, $k_d$ $[M/T^2]$ is the damping constant, $\mathbf {v}_i$ [*L*/*T*] is the *i*-th mass velocity and $\mathbf {r}_{ij}$ is the difference between masses position $\mathbf {r}_i-\mathbf {r}_j$ [*L*]. The numerical units are used in the model: *M*-mass, *L*-length and *T*-time. Here $\mathbf {g}=(0,-5\cdot 10^5)$, $k_s=119755$ and $k_d=365$ (in numerical units as stated above).

The next is the model of pressure. I assume that there is a fluid inside the object with internal pressure $p > p_0$ ($p_0$ is the atmospheric pressure), where the pressure force results from the pressure difference the object boundary. The assumption of constant pressure means that I neglect the dynamics of the fluid inside. To compute the value of pressure inside of the droplet, I use the ideal gas law:

3 $$\begin{aligned} p = nRT/V, \end{aligned}$$

where *p* is the pressure, *R* is the gas constant, *n* is the number of moles, *T* is the gas temperature (I assume it is constant too), *V* is the volume of the droplet (calculated at each step of the simulation). I use Gauss’s theorem and reduce the dimension of the problem by one. For example, to calculate the surface area of the drop in two dimensions I use an integral over the boundary and approximate it as:

4 $$\begin{aligned} S = \int \int _S dS=\oint _l x n_x dl\sum ^{N_L}_{i=1}x_in_{x,i}l_i, \end{aligned}$$

where *S* is the surface of the drop, *dS* is an infinitesimal element on the surface, *l* is the boundary of the drop, $N_L$ is the number of boundary segments, *x* denotes the position in the *x* direction, $n_x$ is the *x* component of the normal vector, *dl* is the infinitesimal element of the droplet boundary, $n_{x,i}$ is the normal vector to the *i*-th boundary segment, $l_i$ is the length of the *i*-th segment.

After computing the surface *S* from equation (4) (or volume *V* in 3D version of the formula), I get the pressure force by using normal $\hat{n}$ to the surface:

5 $$\begin{aligned} \mathbf {f}_p = \frac{nRT}{V} \hat{n}. \end{aligned}$$

Gravity, spring force and pressure forces accumulate at each mass point on the surface. Then, the equations of motion of each point are integrated using the second-order Verlet algorithm. The time step $\delta t=2\cdot 10^{-5}$ is used. After integration, inverse dynamic constraints for the position of the masses are used to stabilize the simulation (Provot 1995). The algorithm 1 in appendix A summarizes all the steps required to implement the model presented in this paper.

## Collisions

I model a porous medium consisting of 2D, random, overlapping and non-overlapping impermeable grains (discs) of varying radius. By varying the number of grains, I control the porosity. For non-overlapping medium, to prevent from touching, the distance between grains was kept smaller than 13$\%$ R (grain radius). For collisions of the soft body with solid grains, I used the penalty method. Each time one of the points at the surface tries to penetrate one of the solid grains, I add a virtual spring with 0 rest length. The spring acts outward on the grain surface. A similar technique was adopted in the static friction model in simulations of granular material before (Risto and Herrmann 1994). Our collision approach is thus similar to the Hertz collision model for soft surfaces.

**Fig. 2:**
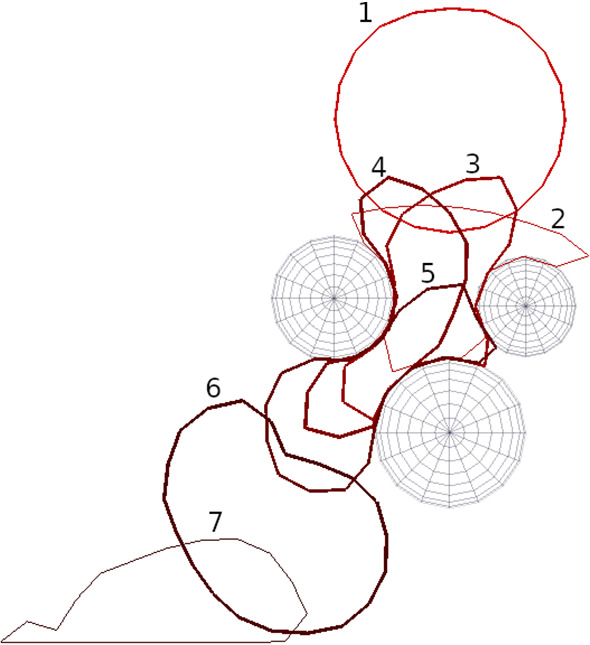
Example simulation of a liquid droplet passing through a single pore formed by three solid grains. Selected time snapshots (ordered by numbers in the figure) of the evolving droplet’s boundary are plotted. The animation of this process is included in the supplementary material as a squeeze.mp4 file

To prevent points on the surface from penetrating further, I move the points out of the obstacle discs and reflect their velocities. This additional procedure was necessary because, occasionally, high droplet velocity and numerical errors related to the integration in the Verlet algorithm caused some points on the droplet surface to get stuck in the obstacles and become blocked. As shown in Fig. 2, the droplet of liquid that starts above the solid grains (initial shape labeled 1) and sinks downward under gravity changes shape on its way into the narrow passage. The whole collision process is presented in the algorithm in appendix A.

## Results

To investigate the relationship between tortuosity and porosity, random configurations consisting of overlapping grains were created. Porosity was controlled by changing the number of grains. At each porosity, 200 independent configurations of grains were used. For each configuration, a maximum of 200 droplets were simulated. The exact number of droplets depended on the number of successful simulations, since droplet plugging could occur in low porosity systems, e.g., due to dead-end pores (Andrade et al. 1997). Therefore, the simulation in low porosity media was time-consuming and may not have converged (the situation where a single droplet stops at a point in the porous region). The initial position of the droplet was randomly chosen in the horizontal direction just above the top of the porous medium.

**Fig. 3:**
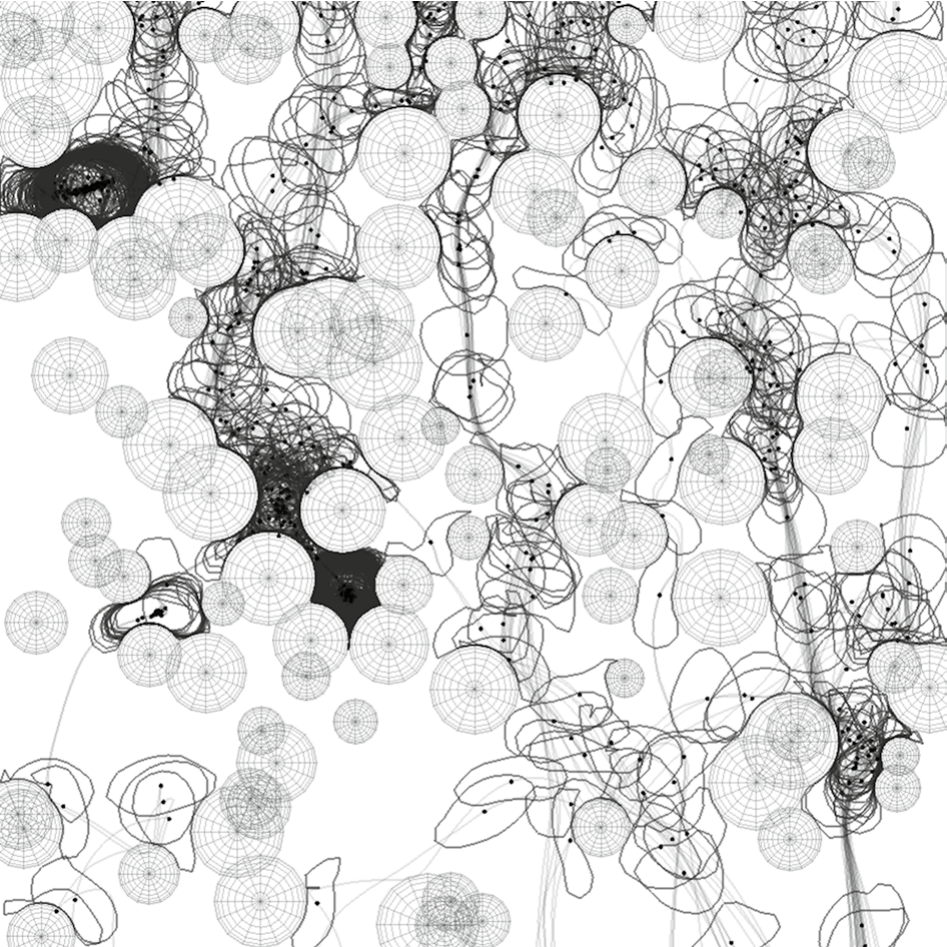
Snapshot of the time course of a 100 single droplet (the center of mass is drawn as a black dot) falling through the porous model of random overlapping disks (wire in the visualization). Also, the path of each droplet that successfully passes through the system is shown as a light gray line. The image uses blending for visualization of multiple simulations

The results of the simulations of falling droplets are shown in Fig. 3. The visualization technique uses alpha blending, where many individual droplet positions are drawn into an image. This allows us to observe regions of increased and decreased droplet presence. If some droplets get stuck, for example, in the dark region in the upper left, it becomes dark because many droplets stay here for a long time. One can notice that some parts of the porous matrix are relatively more permeable. Here, many of the simulations with multiple single droplets pass through this part of the system (e.g., the right vertical channel running from top to bottom, as indicated by the gray path lines).

**Fig. 4:**
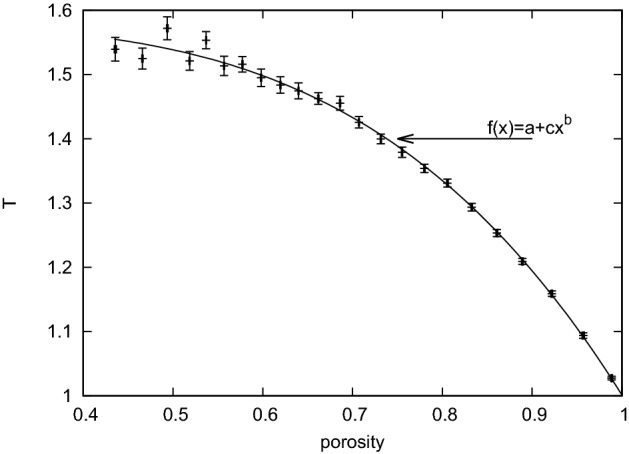
Average tortuosity in the droplet model with varying porosity in the overlapping grains model. Each point represents the average tortuosity, and each error bar is the standard error based on the 200 independent configurations of obstacles. The solid line is the best fit to the function $f(x)=a-cx^b$. I found $a=1.58$, $b=3.87$ and $c=0.58$ using the least-squares algorithm

Using the stored paths for all droplets in all porous samples I simulated, I used the path-based definition of tortuosity (Eq. 1) and calculated the resulting average tortuosity versus porosity, shown in Fig. 4.

By numerically fitting various $T(\varphi )$ relations, I found that the power law:

6 $$\begin{aligned} f(x)=a-cx^b, \end{aligned}$$

fits best (see Fig. 4 caption for results of the fitting procedure).

**Fig. 5:**
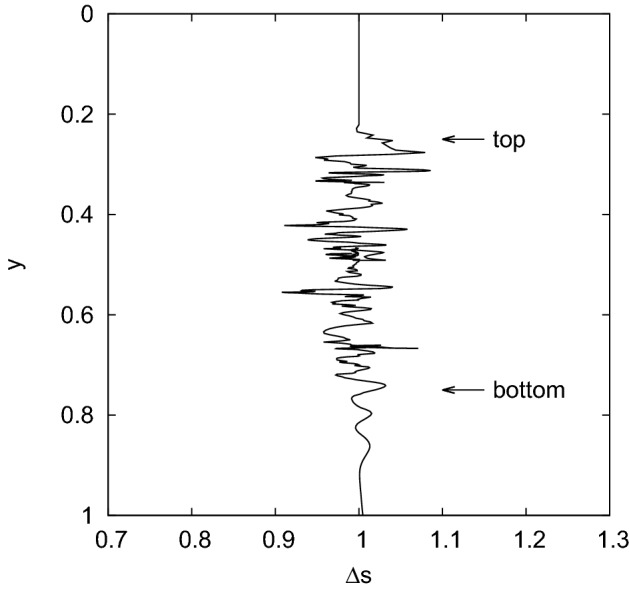
Surface change $\varDelta S$ for the simulation of a single droplet in the porous medium of porosity $\varphi =0.79$ consisting of non-overlapping and slightly separated grains. The arrows indicate the position of the upper and lower interfaces of the medium (the droplet, which was initially above the upper boundary of the medium, was falling through the medium and continued the movement after it came out of the medium due to the flow)

Next, the model was applied to study the motion of a single droplet in a porous matrix composed of non-overlapping grains. Non-overlapping grains were chosen to increase permeability at low-porous media. Inspired by recent experimental and numerical results for a similar process in metal foams (Zhang et al. 2019), I was particularly interested in the changes in droplet volume. Therefore, I observed the change in droplet surface area $\varDelta S=S_p(t)/S_0$. Here, $S_p(t)$ was the area of the droplet in a porous medium that changed over time as the droplet travelled through the porous medium, and $S_0$ was the area of the droplet in the free area (without porous medium). My results are shown in Fig. 5.

I observe a qualitative agreement of our results with simulations and similar experiments reported in Zhang et al. (2019). In my case, the droplet shrinks and expands as it flows through the medium. However, in Zhang et al. (2019), most of the simulations showing the lower bound of $\varDelta S=1$ and $\varDelta S <1$ were performed for a special case of two droplets (one pushed after the other). Modeling two droplets is beyond the scope of this paper, but deserves more attention in the future.

**Fig. 6:**
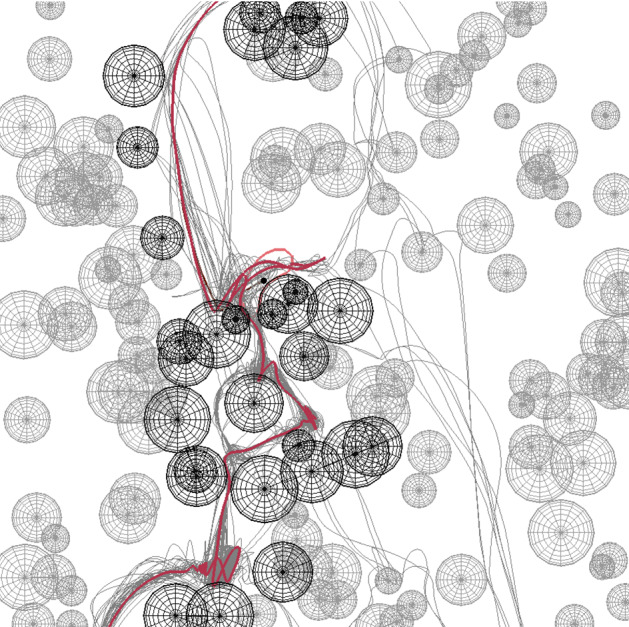
Local search algorithm searches for the configuration of maximum tortuosity in the model of overlapping grains. Here I found the system of $T=2.42$. The solid thick line is the visualization of the longest path. The thin lines represent the set of generated paths with lower tortuosity. The instantaneous configuration with the highest tortuosity is represented by slices visualized as wire spheres. The dark spheres are those that were touched by the droplet in the previous simulation

Finally, I used the model to artificially generate the system at maximum tortuosity. In doing so, I took advantage of the fact that the simulation is very fast, which allowed me to compute thousands of repetitions using a local search algorithm. First, I created a random configuration with a fixed number of grains. Then, I simulated the droplet transport process by starting the droplet above the sample in the middle horizontal position (see Fig. 6).

From this, I determine the tortuosity at the current configuration. After the simulation, I change the position of the grains slightly and run the simulation again. I accept and use the new configuration only if the tortuosity has increased.

To explore the wide range of possible configuration space in the local search algorithm, I slightly and randomly change the position of all grains in the sample. However, at every 10th time step, the special procedure is called. Here, from the list of grains touched by the droplet, I change the position of only one of the grains touched. This directly affects the path of the droplet in the next simulation and lets us explore a natural neighbor solution in the local search algorithm with expected changed tortuosity.

## Discussion

Our results confirm previous findings on tortuosity based on the transport of single-phase fluid flows. The hydrodynamic, single-phase tortuosity grows monotonically with decreasing porosity in the same type, randomly overlapping obstacles, porous medium model (Koponen et al. Jul 1996; Matyka et al. 2008). This suggests that in both single-phase fluids and multiphase single fluid droplets, the largest, most representative pores and channels are chosen as the main channels for transport. In Matyka et al. (2008), the logarithmic tortuosity law

7 $$\begin{aligned} T(\varphi )=1-p\log (\varphi ) \end{aligned}$$

was found to be the best approximation to the model of single-phase fluid flow through an overlapping porous medium with four quads. I found (see Fig. 4), that for droplets, the power law for tortuosity holds:

8 $$\begin{aligned} T(\varphi )-T_0 \propto c\varphi ^b, \end{aligned}$$

where $T_0=1.58$ is the maximum measured value of the averaged tortuosity that is close to the percolation threshold. Here, *c* and *b* are empirical constants that depend on the model. Similar power-law relations between tortuosity and porosity have been found previously, e.g., in Araújo et al. (2006), where $T\propto \varphi ^{-2}$ was proposed for fluid flow through a dilute porous medium consisting of noncontacting (distant) solid grains. None of the above works referred to the motion of individual droplets. In this context, our result is original and it might be worth further investigation to explain why the power law holds in tortuosity–porosity relations between different transport phenomena. Our result may be related to the Archie Law describing electrical and fluid transport through a porous medium:

9 $$\begin{aligned} T=\varphi ^{-b}, \end{aligned}$$

where $b\approx 0.25$ for the model of three-dimensional overlapping spheres (Matyka and Koza 2012). In droplet transport, however, the displacement $T_0$ appears in Eq. 8 which differs from Eq. 9.

**Fig. 7:**
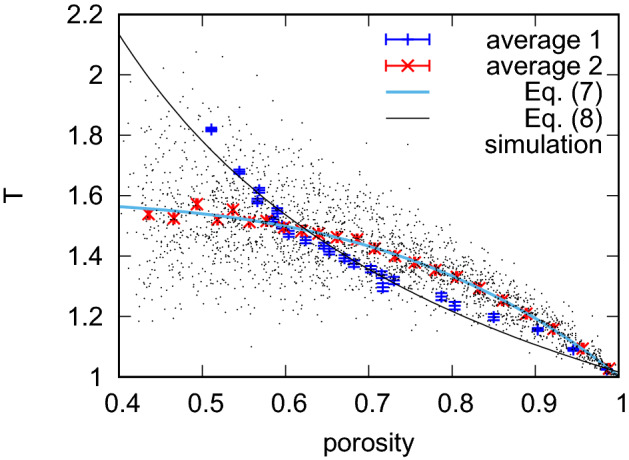
(color figure) Tortuosity of droplets measured in a porous medium with random overlapping grains. Each small black dot represents a porous medium and 200 of independent droplets pushed through the medium. The dot data with the error bars represent two ways of averaging over these data (see text for explanation)

I also noted that different types of scaling can be obtained because of the way the data are averaged. In Fig. 7, I plot the tortuosity data from our simulations (small black dots) and two types of average plots. Here, each point is calculated from 100 points taken from the results with fixed (e.g., considered as the independent variable) tortuosity (average 1) or porosity (average 2). It is interesting to see how these plots differ, especially since they come from the same data set. This shows the importance of proper statistical analysis of scattered data. For example, it is possible that in our earlier work on tortuosity (Matyka et al. 2008) I obtain a simple power law for the hydrodynamic tortuosity if I first sort the original data by porosity or tortuosity. Further analysis of the hydrodynamic tortuosity, while very interesting, is beyond the scope of this paper.

To better understand the role of specific forces in droplet dynamics, I estimated the Bond number for the model using the following definition:

10 $$\begin{aligned} \mathrm {Bo}=\frac{\mathbf {g}\varrho L^2}{\sigma }, \end{aligned}$$

where $\mathbf {g}$ is the gravity, $\varrho$ is the fluid density, *L* is the typical length scale and $\sigma$ is the surface tension of the droplet (Hager 2012). I start with the analysis of the free-falling drop without deformation. The surface tension is calculated from the force along the interface taken from Hooke’s spring properties (see equation) $\sigma =-k_s\cdot \delta x+k_d\cdot \delta v=199$ $[M L / T^2]$ (*M*—mass, *L*—length, and *T*—time units, respectively). I used spring $k_s=20\cdot 10^3$ $[M / T^2]$ and damping constant $k_d=3.6\cdot 10^2$ $[M / T^2]$ for all segments on the surface. Gravity and density were $\mathbf {g} = 3.4\times 10^4$ $[L/T^2]$ and $\varrho =$mass/volume$=20/0.0063=3175$ $[M/L^2]$ (all data in simulation units). The characteristic size was chosen as the initial radius of the droplet $L=0.03$ [*L*]. Thus, at equilibrium, Bo$=488$. Such a large number indicates that our droplet is not affected by surface tension and gravity dominates. However, if we consider a droplet deforming in a porous medium, the situation changes. My calculations show that Bo there varies between 2.1 and 1365, which is mainly due to strong variations in the surface tension ($\sigma =25453$ and $\sigma =0.07$ $[M L / T^2]$, respectively) due to the stretching and squeezing of the droplet in the pores.

Technically, from the droplet model point of view, one of the main problems I had during the simulation was the mobilization of the trapped droplets in the low-porosity systems. Therefore, I decided to change the model for simulating the pushing of droplets in low-porosity systems and studying surface changes from an overlapping to a non-overlapping model. This greatly improved the simulation. In the future, one might also consider including some sort of vibrational mechanism for dealing with trapped droplets (e.g., vibrations Li et al. 2005), which could easily be implemented in the presented model.

## Conclusions

In this paper, I presented the new results for tortuosity based on the motion of a single drop of fluid simulated with a soft body pressure mechanical model. My results show agreement with basic power laws for tortuosity, qualitatively agree with observations based on tortuosity indices for single-phase flows and open new perspectives for further research in this field, since quantitative agreement is still an open question. The new concept of multiphase tortuosity presented in this work could be used to formulate new empirical laws for multiphase permeability, since I can expect that the more wobbly, blocked and complex the pore space is (in terms of droplets), the lower the permeability of the system will be.

There are several problems where the motion of individual droplets plays an important role and, after some improvements, can be simulated with the model presented in this work. However, there is still work to be done on validating the model with experiments, comparison with the solver for multiphase liquids, wetting, splitting and bonding of a droplet, the third dimension and the study of arbitrarily shaped particles, which I leave to further research.

### Supplementary Information

Below is the link to the electronic supplementary material.

Supplementary Information (744 KB)

## Data Availability

Not applicable—the data are included in the manuscript as results in plots and figures.

## A Algorithm Used in the Simulation of Droplets

For the simulation, I used the spring–mass softbody model with pressure force (Matyka and Ollila 2003) in two dimensions. The algorithm starts with initializing the geometry and constants in lines 1-2 (spring constant $k_s$, damping constant $k_d$, gravity $\mathbf {g}$, gas mole *n*, gas constant *R* and temperature *T*, $\varDelta t$ numerical time step. I used the following values for the simulations to simulate the dynamics of the droplet in Fig. 3: $k_s=19755$, $k_d=365$, $\mathbf {g}=(0,-34\cdot 10^{3}$), $nRT=20\cdot 10^{2}$, $\varDelta t=2\cdot 10^{-5}$. A relatively large numerical value for gravity was used to effectively push the droplet through the system. Accordingly, a small value for $\varDelta t$ was needed.

I initialize the geometry of the object in line 2. In the 2D case, points are evenly distributed on the edge of the circle and connect all closest neighbors with springs so that the model forms a closed loop. Then (lines 3–7) I compute the rest lengths $d_0$ for each spring.

The simulation starts (line 8) and runs until $t< t_{end}$. First, gravity (lines 9–11), spring force (12–18) and pressure force (20–24) are accumulated for each point. The pressure force is preceded by the calculation of the surface area in line 19 (note that this should be the volume if the model works in 3D). Since our model is supposed to work in a porous region consisting of overlapping random slices, I check the collision of the droplet with the porous geometry (lines 25–32). Here I use a twofold procedure in which, for each point on the surface of a soft body that penetrates an obstacle, I add the force pulling out of the disc and, in addition, move the point outward and reflect its velocity. The method prevents collisions and sticking of the droplet to the geometry at large velocities that occur in our system.

I update the positions of all points using the Verlet algorithm (lines 33–35) and limit the possible deformations of the surface springs using inverse dynamics constraints (Provot 1995) up to half of the rest length (lines 36–38). Finally, I collect data about the position of the droplet, compute its center of mass, compute path tortuosity and continue simulation until necessary (lines 39–40).

## Code availability (software application or custom code)

Not applicable—the article consists of complete model algorithm.
